# A Practical Treatise on Venereal Diseases

**Published:** 1841-07-01

**Authors:** 


### A COMPLETE PRACTICAL TREATISE ON VENEREAL DISEASES, AND THEIR IMMEDIATE ARD REMOTE CONSEQUENCES. INCLUDING OBSERVATIONS ON CERTAIN AFFECTIONS AND DISCHARGES. By *William Acton*, late Externe at the Female Venereal Hospital, Paris. Octavo, pp. 410 London, Renshaw, 1841.

MR. ACTON has been a zealous and ardent disciple of M. Ricord's. He has followed his practice, collected his opinions, become imbued with his ideas, and laudably endeavours, in the present volume, to put the English reader in possession of them. The following dedication sufficiently expresses Mr. Acton's sense of what he owes to M. Ricord. To that gentleman it is addressed.

" Europe may admire the genius of the author of the 'Traité Pratique sur l'Inoculation,' the British critic may call you the French Hunter, and the Institute may crown you with its laurels; but the pupil who, for a series of years, has watched you at the bedside, performing the varied experiments which are overturning long-received opinions, and collecting those facts which form the basis of a new school, can alone adequately feel, and sufficiently appreciate, that honest and manly candour which is always ready to acknowledge errors, and that generosity which allows others to participate in opinions still unpublished."

As we purpose, on a future occasion, returning to M. Ricord's work and to this, we shall content ourselves for the present with noticing incidentally a few points of practice.

### *Diagnosis between a virulent Gonorrhœa and a simple one.*

The French surgeons have a great objection to the term *gonorrhœa*. They point to its derivation and absurdity. But it has been so long in use, and the ideas derived from its origin are so exploded, that it matters little whether we stick to it or *blennorrhagia*. Be that as it may, there has been, to our minds, a great deal of useless altercation on the means of distinguishing a "virulent" gonorrhœa from a "simple" one. Indeed it is difficult to know exactly what a "virulent" gonorrhœa is. Is it a severe one, or one followed by secondary symptoms? We apprehend that different authors will be found to use the term in different ways. The following are M. Ricord's views.

" The previous views of authors show how much difference of opinion existed on this subject when M. Ricord undertook to show that the cause of a virulent blennorrhagia depended upon the complaint being complicated with a *chancre*. In women, more especially, he found that what was called a virulent gonorrhœa depended upon the existence of ulcerations, which could not be discovered by an examination of the external organs of generation, but which the use of the speculum clearly proved to exist; but did all ulcerations give rise to a virulent gonorrhœa? was the next question to be solved. At the time this eminent surgeon was investigating the subject, he often had occasion to treat the woman from whom some of his male patients had contracted the disease, and he found that there were various forms of ulcerations, the secretion of which caused simply a mild gonorrhœa: there existed others, however, which caused sometimes chancres on the glans penis and prepuce; on other occasions, virulent gonorrhœas. In vain did he try to distinguish these ulcerations by their physical characters. It was only by inoculation that he was enabled to prove why sores similar in appearance gave rise to such different consequences. Inoculation soon showed him that there may exist an ulceration of a *specific* character, which will be described in its proper place, and called chancre; but there likewise exist ulcerations of a *simple nature,* the result of an inflammatory state of the mucous membrane, which were frequently the consequence of a blennorrhagia. From this moment that which was previously doubtful became clear, and an inquiring and observing mind like his was not long in deciphering what had been the *opprobrium medicorum.*

From what the speculum showed clearly to exist in the vagina, he naturally concluded that similar appearances might exist in the urethra of the male, but which, from its small size, it was impossible to demonstrate. However, one opportunity of examining the urethra, followed soon after by a second, put him in possession of two cases, which he showed to the Academy of Medicine, in which chancres existed in the whole course of the urethra. He thus discovered the key to this hitherto difficult labyrinth; and concluded that the only diagnosis between virulent and mild blennorrhagia is derived from inoculation.

The experiments, frequently repeated, of inoculating with the secretion of a simple mild blennorrhagia, produce only a slight irritation, which subsides in a few hours; whereas, if the complaint is virulent, or, in other words, depends upon, or is complicated with, a chancre which is concealed, or which can be brought into view by the speculum, the secretion introduced under the skin, in a similar way as in the former experiment, will produce a vesicle, pustule, and chancre. This, then, I call the *certain pathonomonic* diagnosis of a virulent blennorrhagia. A rational diagnosis may be drawn from the rosy, thin, serous, or rusty colour of the discharge, provided such be present, as well as from an indurated spot in any point of the canal, accompanied with fixed pain, &c.

Should buboes follow, which on inoculation give rise to the characteristic pustule, it may be asserted confidently that the blennorrhagia is a virulent one. The occurrence of secondary symptoms, which only follow in a few cases, gives a further diagnosis of the same fact.

It is, however, the rational diagnosis that the surgeon must usually depend upon, as inoculation cannot always be proposed, or he may find patients object to submit to it; he must, however, remember that it is but a rational one, and on such data be cautious how he risks his reputation by giving an opinion." 47.

That there are cases of syphilitic sore in the urethra is indubitable. We have ourselves published two or three, and seen two more. In those cases the sores have been easy of detection by any observant surgeon. The inoculation test is a favourite one with M. Ricord. We confess that we think the value of inoculation in cases of venereal disease is overrated. First, fallacies may stick to it. In a bad habit of body any discharge might produce by inoculation a sore, and one of ugly characters. What sort of evidence would that be? Suppose (what we believe has happened) a phagedænic sore so produced. Is that an evidence of virulence or not? It is neither, because, phagedæna, depending on the state of the recipient rather than on the nature of the virus, syphilitic matter may occasion it as well as other matter. Short of phagedæna, we have no doubt in our own minds that discharges of very different kinds might give rise by inoculation to sores of dubious character.

In the second place this difficulty has always seemed to us to attach to inoculation. A sore has a doubtful character. To determine if it be syphilis we inoculate with it. Suppose a similar sore produced. It does not follow that that is syphilitic. Acrid discharge occasioning the first may occasion the second. If syphilis had as fixed characters as vaccinia or as variola the discrimination would be easy. The standard of comparison being precise, the object selected could fairly be compared with it. But if the standard be a fluctuating one, what strict comparison can be instituted ? If, indeed, only syphilitic sores could be communicated by inoculation, the difficulty would vanish. But this we disbelieve in toto. Look at the effect of dissection wounds, at the effect of dabbling in acrid discharges totally different from venereal ones. Sores are excited that, occurring on the penis, or produced by inoculation with gonorrhœal matter, would, not unfairly, be pronounced syphilitic.

In the third place, it is by no means certain that genuine syphilitic matter must of necessity reproduce a sore. It would require a vast array of experiments to establish this. A person may be inoculated with variolous matter and not take variola, with vaccine matter and not exhibit the vaccine vesicle. In the act of connection, a brief contact with syphilitic matter causes syphilis. Yet the discharge from that syphilitic sore may lodge for weeks upon the prepuce and not generate a second sore. Why may not inoculation be inoperative too ?

Finally, inoculation is in practice useless, or not very remote from useless. What surgeon in this country would propose, what patient submit to being inoculated with matter from his urethra, to solve a difficulty more apparent than real, and very likely not to be solved after all ? What surgeon would propose, what patient submit to, the risk of phagedæna for such a reward ? We say phagedæna, for we are confident that were inoculation general, phagedæna would every now and then occur. A French hospital and an English consulting room are very different places.

Our own conviction is that inoculation has been overrated—that it is much less satisfactory and much more objectionable than is represented.

*Is the Speedy Suppression of a Gonorrhœa safe ?*

"A patient will sometimes ask the surgeon if the treatment he is about to prescribe will give rise to a stricture or a swelled testicle. It is a very common error to suppose that the treatment will occasion either of these complaints, and this, like many other popular errors, has taken its source in medical writings, which have stated that a blennorhagia speedily cured will give rise to various other complaints. M. Ricord says, we may state distinctly that no ill consequences are to be feared from any treatment, provided it is not grossly improper. If (pursues that surgeon) I were disposed to be aphoristic, I might say that the ill consequences will be few, in proportion as the cure is speedy ; and I defy any one to produce a case cured in twenty-four hours from its commencement, which has been followed by any ill consequences. There are prejudices against speedily curing a blennorrhagia, and I may be told by some surgeons, 'after a practice of full thirty years, I am of an opposite opinion.' But I ask, may not such a practitioner have laboured under a mistaken notion during thirty years ? Is implicit belief in a fact, for that space of time, a proof that that fact is true ? Has not an old author said, and very truly, 'experientia fallax ?'" 48.

It is odd enough that this very day a practical comment on this doctrine came before us. A gentleman whom we had treated for a gonorrhœa two or three years ago, and that with safety and success, applied to us under these circumstances. Last year he went to Paris, and caught a gonorrhœa there. He went immediately to M. R——, who gave him very powerful doses of capivi and cubebs, and strong injections. He felt, he said, all on fire with the medicines. The gonorrhœa was stopped in a week, but from that time to this he has been subject to severe headaches, his digestive organs have been totally deranged, and a stricture soon supervened. We have no hesitation in expressing our dissent from the doctrine that M. Ricord has laid down.

### *Diagnosis of Gonorrhœa.*

"Every tyro in medicine will at once distinguish what he calls a clap, by means of the symptoms above described: but such a person may not be aware that a surgeon cannot always decide at once whether a man is suffering under a gonorrhœa or not, provided no discharge be observed, and the lips of the urethra be not inflamed, and no stains seen on the linen. M. Ricord gives the following instance of the occasional difficulty. He was ordered by a magistrate to give an opinion whether or not a prisoner, said to have violated a girl, was labouring under gonorrhœa or not. The accused presented no swelling of the lips of the meatus; on pressure, no discharge came from the urethra, and there existed no traces of any secretion on the shirt. When interrogated, he stated he had made water six hours previously to the examination. As M. Ricord had some suspicion, he ordered him to pass his urine at once, and desired one of the gaolers to watch his prisoner; in six hours after, M. Ricord returned, and then found undoubted marks of an existing gonorrhœa; the prisoner confessed that he had made water previously to the first examination, and had taken care to remove the secretion as soon as formed by a piece of lint which he concealed for that purpose." 75.

We have never seen an "undoubted" gonorrhœa without an obvious vascular fulness of the mucous membrane. On everting the lips of the urethra, it is either seen florid, with a punctuated redness (we employ a lens) and a semi-abraded appearance, as if the epithelium were partially removed, or the veins of the mucous membrane are enlarged and tortuous.

### *How to compress an Inflamed Testis.*

"The *emplastrum vigo cum mercurio*, of the French codex, is the preparation that M. Ricord employs, cut into strips about half an inch wide; but other adhesive plasters answer equally well; the less irritating they are, however, the better.

The manner of employing them is as follows; embrace the affected testicle in your closed hand, drawing it, at the same time, away from the other; then pass a strip of plaster around the chord, just where it is in connexion with the testicle, to prevent the testicle escaping out of the scrotum; this being done, the scrotum of the affected side will be applied closely on the testicle, which presents an oval shape; the strips of plaster may then be applied in circular layers along the testicle, until all but the lower part is included. The latter may then be compressed by smaller and shorter strips, placed at right angles with the circular ones, which they thus maintain in their place. The testicle is thus equally compressed, and the strips of plaster should be drawn tolerably tight; the patient will usually complain of some degree of pain during and immediately after the operation.

Far from discouraging the surgeon, this should lead him to believe that the compression is well applied. When, however, the pain has not abated at the end of an hour, the surgeon may usually be assured that no good can result from this treatment; the compression should be discontinued, and antiphlogistic means had recourse to: if the cases of compression be well selected, and the indications followed, it will be necessary to withdraw the plaster in a few hours after, for the size of the testicle will have subsided considerably, and the shell of adhesive plaster will become quite lax: it must be removed, and other strips applied in the same manner as the former, which may require removal in succession." 102.

We confess that this plan, which has been in some vogue on the Continent, is one that we should hesitate to recommend.

*Disease of Cowper's Glands.*

Our author observes that during the course of gonorrhœa, it not very unfrequently happens that we find an affection of Cowper's glands come on, and this affection is more common than is generally supposed; it may come on imperceptibly, and the patient take no notice of it until there is considerable swelling of the parts. In other instances there is fever, and all the symptoms of abscess; pain is felt in the course of the urethra, followed by fluctuation at one point, and difficulty in making water. M. Ricord considers that the affection commences in Cowper's glands in consequence of the extension of inflammation, and that suppuration results, and has a tendency to make its way outwards.

An abscess, however, may occur in the course of the urethra, in consequence of an abrasion of the mucous membrane, and a limited infiltration of urine follow; the abrasion may heal, and a small abscess will result situated close to the urethra; such a case it will be impossible to distinguish from inflammation of Cowper's glands, if it occurs in or about the bulb. To the finger this abscess will give the sensation as if it were attached to the urethra by a pedicle.

*Passion of French Ladies for the Speculum.*

"M. Ricord states that the French female in high life is now so reconciled to the use of the speculum, that he often receives notes requesting his attendance, and, in the postscript, a demand that he will bring his speculum. At the hospital, when M. Ricord took the service in 1830, a revolution broke out, and *a strike* against the speculum occurred; however, a few days of bread and water quieted this revolt of the harem, and now the women are so convinced of the benefit, that they think no more of its introduction than they do of having a blister dressed. They can, however, appreciate instantly the tact of one surgeon over the other in introducing it." 180.

Certainly this latter is an enviable distinction, and one that a surgeon may be proud to earn. Mr. Acton anticipates the period when the absurd objections now entertained by English ladies will yield, and like the French, they will send a note to the Doctor, with a P. S. (notoriously the portion of a lady's letter in which she speaks her mind) for a speculum. Mr. Acton anticipates this, and that "time will overcome the prejudices which exist" against the instrument. We confess that we are among those who hope that these "prejudices" will always be characteristic of our countrywomen, and that it will be reserved for the most abandoned class to *request* the introduction of the speculum. Mr. Acton is partial to it, for he gives an engraving of a "speculum chair," which he recommends as ornamental to a library.

*The Certificate System.*

Rather an amusing insight into the state of society in France is afforded by the different forms of certificate given us (after Ricord) by Mr. Acton. We will quote one as a sample. They are in blank, to be filled up with name and date.

"After carefully examining Mrs. ———, I do not find she is labouring under any syphilitic affection, but is subject to a discharge, which, under the influence of any exciting cause, may become aggravated, and thus transmit the disease to persons having connexion with her." 184.

We have never heard that this sort of pass-port system obtains in this country. Unhappily, the work before us offers other indications of the loose morality of Paris. Some of the recommendations and cases of M. Ricord we forbear to introduce. The following fact is a painful one.

"During the period I performed the duties of Externe, under Professor Velpeau, at La Charité, a mother brought into the hospital a little girl of three years of age, affected with a white swelling and a discharge from the vagina. She stated that the infant was in the constant habit *de s'amuser*, as she called it, and when left alone, repeated continually this mal-practice; she further traced the habit, so early commenced, to a plan which nurses in France have of tickling the genital organs of children who are peevish; this, for the moment, quiets them, but infants repeat these manipulations even at a very early age, as this case proves. On inquiry I found that this was not an isolated case, and leads in after life to most vicious propensities." 27.

Before closing this volume we must repeat that it offers a complete account of the views and practice of M. Ricord. Any one who peruses it will see in it the merits and the faults of the French School. The author deserves credit for the manner in which he has executed his task, and he shews that his time has been diligently spent in the French hospitals. But we think that, in his laudable anxiety to enhance the reputation of his preceptor, he fails to do justice to what is known in this country.

